# Multipoint pacing for cardiac resynchronisation therapy in patients with heart failure: A systematic review and meta-analysis

**DOI:** 10.1111/jce.15199

**Published:** 2021-08-19

**Authors:** Vishal S Mehta, Mark K Elliott, Baldeep S Sidhu, Justin Gould, Bradley Porter, Steven Niederer, Christopher A Rinaldi

**Affiliations:** 1 Department of Cardiology, Guy’s and St Thomas’ NHS Foundation Trust, London, UK; 2 Department of Imaging Sciences and Biomedical Engineering, King’s College London, London, UK

**Keywords:** cardiac resynchronisation therapy, efficacy, meta-analysis, MPP, multipoint pacing, systematic review

## Abstract

**Introduction:**

Multipoint pacing (MPP) has been proposed as an effective way to improve cardiac resynchronisation therapy (CRT) response. We performed a systematic review and meta-analysis evaluating the efficacy of CRT delivered via MPP compared to conventional CRT.

**Methods:**

A literature search was performed from inception to January 2021 for studies in Medline, Embase and Cochrane databases, comparing MPP to conventional CRT with a minimum of 6 months follow-up. Randomised and nonrandomised studies were assessed for relevant efficacy data including echocardiographic (left ventricular end systolic volume [LVESV] and ejection fraction) or functional changes (New York Heart Association [NYHA] class/Clinical Composite Score). Subgroup analyses were performed by study design and programming type.

**Results:**

A total of 7 studies with a total of 1390 patients were included in the final analysis. Overall, MPP demonstrated greater echocardiographic improvement than conventional CRT in nonrandomised studies (odds ratio [OR]: 5.33, 95% confidence interval [CI]: [3.05–9.33], *p* < .001), however, was not significant in randomised studies (OR: 1.86, 95% CI: [0.91–3.79], *p* = .086). There was no significant difference in LVESV reduction >15% (OR: 1.96, 95% CI: [0.69–5.55], *p* = .20) or improvement by ≥1 NYHA class (OR: 2.49, 95% CI: [0.74–8.42], *p* = .141) when comparing MPP to conventional CRT. In a sub analysis, MPP programmed by widest anatomical separation (MPP-AS) signalled greater efficacy, however, only 120 patients were included in this analysis.

**Conclusion:**

Overall MPP was more efficacious in nonrandomised studies, and not superior when assessed in randomised studies. There was considerable heterogeneity in study design making overall interpretation of results challenging. Widespread MPP programming in all CRT patients is currently not justified. Further large, randomised studies with patient-specific programming may clarify its effectiveness.

## 1 Introduction

Cardiac resynchronisation therapy (CRT) is an effective treatment for patients with heart failure (HF) and electrical dyssynchrony characterised by left bundle branch block (LBBB), however, between 30% and 50% fail to respond. CRT nonresponse is multifactorial, however, placement of the left ventricle (LV) pacing lead away from scar may play a significant role in determining response.^1^ Multipoint pacing (MPP) is achieved when multiple pacing stimuli are delivered via a single quadripolar lead placed in a branch of the coronary sinus to achieve CRT.^2^ Early feasibility and single-centre studies demonstrated positive results with improvements in acute and short-term measures of dyssynchrony and haemodynamic response.^3–5^ Determining efficacy of MPP is essential as this technology has been demonstrated to reduce battery longevity.^6^ Small randomised studies,^7^ and a nonrandomised prospective registry^8^ has demonstrated significant symptomatic and echocardiographic improvements when comparing MPP to conventional CRT. Subsequently the MPP trial by Niazi et al.^9^ demonstrated noninferiority of MPP to conventional CRT with significant benefit in a nonspecified subgroup analysis of maximally separated programming (MPP-AS). More recently, the MORE-CRT MPP study by Leclercq et al.,^10^ failed to demonstrate benefit of MPP compared to CRT in terms of volumetric response, however, it did show improvement in a non prespecified subgroup with MPP-AS programming. A prior meta-analysis evaluating MPP included 11 studies and concluded that MPP reduced HF hospitalisation (odds ratio [OR]: 0.41, 95% confidence interval [CI]: [0.33–0.5], *p* < .001), increased CRT response (OR: 3.64, 95% CI: [1.68–7.87], *p* = .001) and decreased cardiovascular mortality (OR: 0.21, 95% CI: [0.11–0.4], *p* < .001).^11^ The authors concluded that MPP was more effective than standard CRT, however the meta-analysis included studies which did not only employ MPP, but rather the effects of single site stimulation from a quadripolar lead. Since this prior metanalysis, two further major randomised trials evaluating MPP have been published^9,10^ and a meta-analysis incorporating the data from these randomised trials is needed to evaluate the utility of this treatment.

## 2 Methods

### 2.1 Literature search

The systematic review and meta-analysis were conducted in accordance with the PRISMA (Preferred Reporting Items for Systematic Reviews and Meta-Analyses) guidelines for all stages of the design and implementation (see Table S1).^12^ We systematically reviewed the relevant literature, comparing MPP to conventional CRT, by searching EMBASE, CENTRAL, and MEDLINE databases from inception to January 2021 without language restriction. The Quality of Reporting of Meta-Analyses statement^13^ and the empiric study by McAuley^14^ indicate the exclusion of unpublished studies produces a systematic positive bias, and therefore “grey literature” in the form of poster presentations, unpublished data from Cochrane reviews or other meta-analyses, conference abstracts and preprints were included. In addition, references of review articles, meeting abstracts, pre-prints, letters, editorials, and previous meta-analyses were searched. The robustness of the decision to include “grey literature” in the final analysis will be tested by a sensitivity analysis. The following keywords were used for search: “multipoint pacing” OR “multipoint left ventricular pacing” OR “Multi-point pacing” OR “multi-point left ventricular pacing” OR “MPP pacing.”

### 2.2 Selection criteria

We included all eligible studies that met the following inclusion criteria: (i) CRT-Pacemaker/Defibrillator (CRT-P/D), (ii) MPP (defined as two or more pacing sites from a single quadripolar lead) versus control group (i.e., conventional CRT), (iii) human studies only, (iv) minimum of 6 months mean follow-up. We excluded studies that only assessed acute haemodynamic and/or electrical metrics. For each included study, data of the following efficacy endpoints were used and evaluated: (i) echocardiographic volumetric response (change in ejection fraction (EF) or left ventricular end systolic volume [LVESV]), and symptomatic response (change in New York Heart Association [NYHA] class or Clinical Composite Score [CCS]). Observational and randomised trials were included.

### 2.3 Data extraction

All data from included studies were independently extracted and assessed for further analysis by two reviewers (CAR and VSM). Any discrepancies were resolved through a third reviewer (MKE). From each study, relevant information regarding reported baseline patient characteristics (age, aetiology of HF, QRS duration), follow-up time, and baseline prespecified efficacy endpoints (LVEF, LVESV) were extracted and tabulated. Characteristics of studies, including study design, number of patients included in the analysis, inclusion criteria, primary and secondary efficacy endpoints, and MPP configuration, are reported. MPP configuration was defined as: (i) MPP-AS: study participants programmed to anatomically separated MPP, (ii) MPP-Other: study participants programmed by implanter preference, (iii) MPP-Mixed: study participants programmed to either MPP-AS or MPP-Other.

### 2.4 Data synthesis, sensitivity, and statistical analysis

Intention-to-treat data were used for evaluating clinical endpoints from included studies whenever possible. It was anticipated that there would be considerable variation in the efficacy endpoints reported, therefore an echo volumetric endpoint (either reduction in LVESV >15% or increase in LVEF >5%) and clinical improvement endpoint (either CCS or NYHA class improvement by 1) was considered. The OR and respective 95% CIs were computed for categorical variables (study pooled echo volumetric and clinical endpoints, reduction in LVESV >15%, and improvement in NYHA class or CCS) and as appropriate, the standardised mean distance (SMD) and respective 95% CIs were evaluated for continuous variables (LVESV). As an aid to interpretation of the results, we conducted a sensitivity analysis to assess the role that inclusion of “grey literature” had on observed results and to evaluate the robustness of the inclusion criteria. To estimate prespecified efficacy endpoints of continuous data, only those publications that contained both baseline and follow-up means ± *SD*s were used. Significant heterogeneity was expected, and so a random effects meta-analytical approach was applied to all analyses. Heterogeneity was assessed by calculating τ^2^ and I^2^, and was considered low, moderate, and high for I^2^ values of <30%, 30%–60% and >60%, respectively.^15^ Sub-group analysis according to the study design and MPP pacing configuration versus conventional CRT were performed. Analyses were performed using R version 1.3.1093 with the “metafor” package.^16^ A significance level of 0.05 was used for testing and confidence intervals, and all testing was two-tailed. Risk of bias for the individual studies was performed with the Cochrane Risk of Bias 2 tool and presented graphically.^17^ As less than 10 studies were included in the final analysis, no test for funnel plot asymmetry was performed.^18^

## 3 Results

A total of 203 unique records were identified through the database and bibliographic searches. Of these, 151 were excluded based on title and abstract content. After screening the full texts of the remaining 52 articles, 7 met inclusion criteria (Figure S1). A total of 5 randomised controlled trials (3 multicentre) were included, of which 3 had an active run-in period of conventional CRT for a period of at least 3 months, and 2 did not. A total of 2 were observational registry analyses, of which 1 was prospective, and 1 was retrospective. A total of 5 were fully published results,^7–10,19^ 1 was an abstract,^20^ and 1 was a full-text preprint of an earlier published abstract^21,22^ (Table 1). In total 1390 patients were included in the final analysis (669 undergoing MPP, and 721 undergoing conventional CRT). In the MPP programming sub-analysis, 519 patients were included (217 undergoing MPP-AS, 205 undergoing MPP-Other, and 97 undergoing conventional CRT). All patients had either a Unify Quadra MP™ or Quadra Assura MP™ (St Jude Medical) CRT device implanted along-side a Quartet™ quadripolar LV lead (St Jude Medical). Mean follow-up ranged between 6 and 26.5 months (Table 1). Baseline characteristics of study participants are reported in Table 2. Graphical risk of bias assessment is reported (Figure S2).

### 3.1 Primary efficacy endpoint and study design sub-analysis

**Figure 1:**
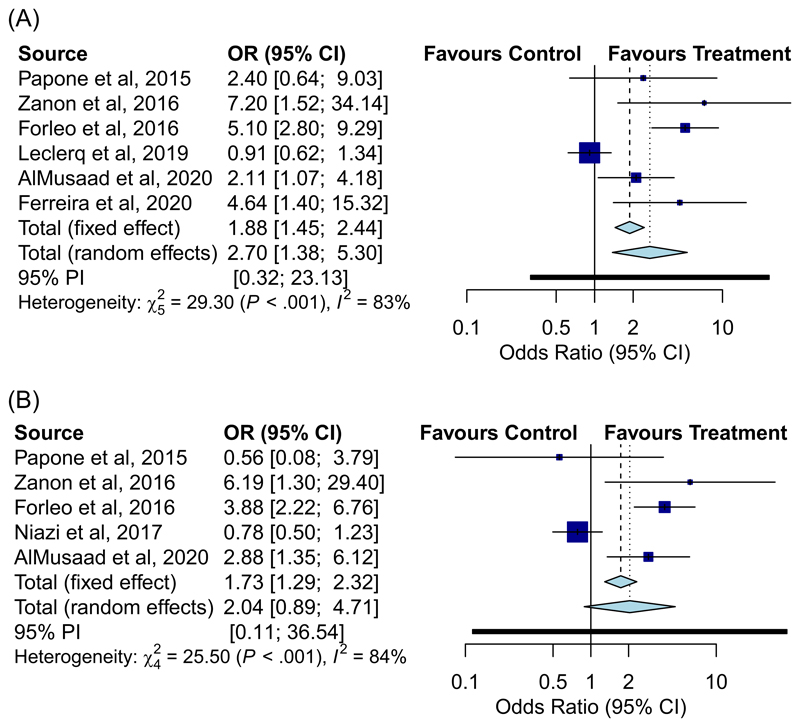
Forest plot displaying odds ratio of achieving echo volumetric (A) and clinical response (B) in all studies

**Figure 2:**
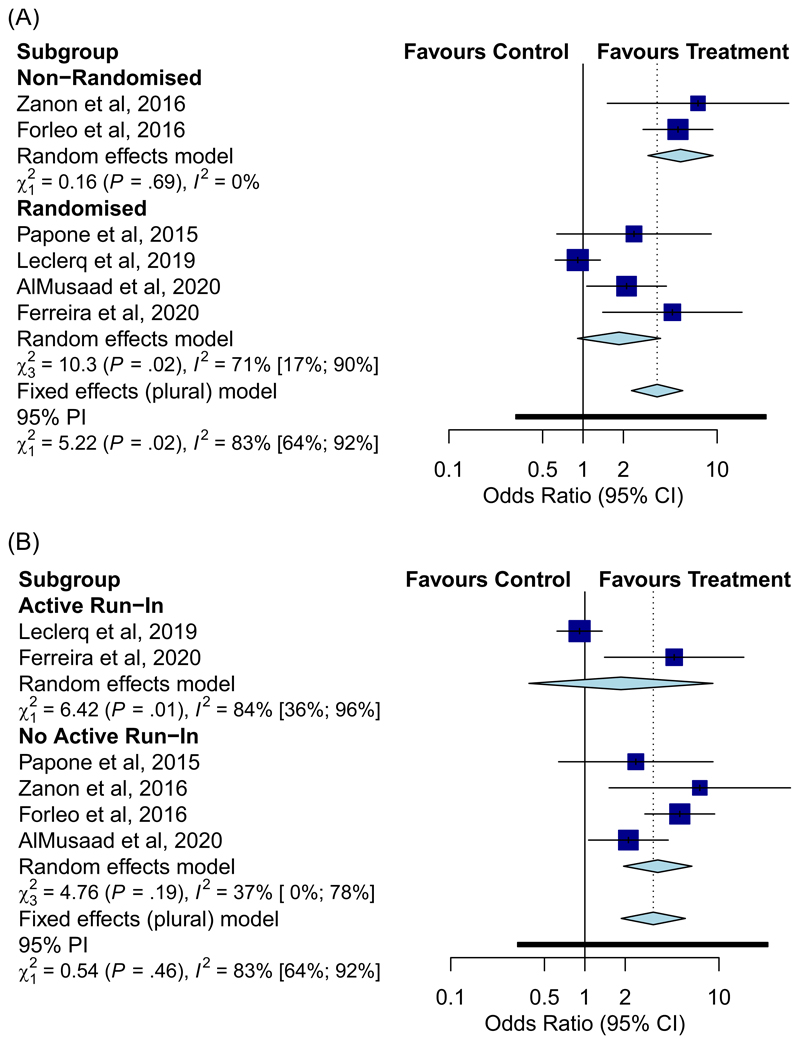
Forest plot displaying odds ratio of achieving echo volumetric endpoint dependent on study design. Randomised vs Non-Randomised (A) and Active run-in v No Active run-in (B)

Overall, MPP was found to be more effective than conventional CRT (OR 2.70, 95% CI: [1.38–5.30], *p* = .004; I^2^ = 83%] of achieving an echocardiographic improvement, and only trended towards significance of achieving functional improvement (OR: 2.40, 95% CI: [0.89–4.71], *p* =, I^2^ = 84%) (Figure 1). Sub-group analyses with regards to echocardiographic response were performed by study design: (i) randomised versus nonrandomised, and (ii) with active run-in versus without active run-in (Figure 2). MPP was effective in the nonrandomised analysis [OR: 5.33, 95% CI: [3.05–9.33], *p* < .001; I^2^ = 0%] and not-effective in the randomised analysis [OR: 1.86, 95% CI: [0.91–3.79], *p* = .086; I^2^ = 71%]. 3 studies had an active run-in of at least 3 months with conventional CRT before randomisation^9,10,20^ of which 1 study by Leclercq et al.,^10^ only randomised CRT non-responders after 6 months. Studies with an active run-in demonstrated no significant difference in efficacy of MPP [OR: 1.86, 95% CI: [0.38–9.01], *p* = .442; I^2^ = 84%) whereas those without an active run-in did demonstrate MPP efficacy (OR: 3.50, 95% CI: [1.85–6.28], *p* < .001; I^2^ = 37%).

### 3.2 Sub-analysis by programming type

**Figure 3:**
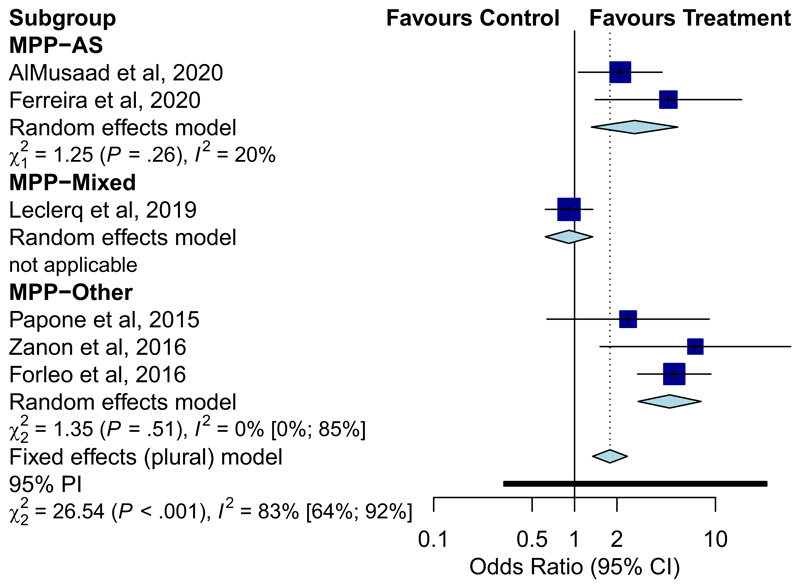
Forest plot displaying odds ratio of achieving echo volumetric endpoint of MPP programming type versus conventional CRT. CRT, cardiac resynchronisation therapy

A sub-analysis was performed of studies depending on type of MPP programming (as defined above), in the participants randomised to MPP vs conventional CRT. Two studies compared MPP-AS only,^20,22^ and two compared a combined cohort of MPP programming (MPP-Mixed),^9,10^ to conventional CRT. Three studies did not specify degree of final programming separation (MPP-Other).^7,8,19^ Of those, Zanon et al.^19^ optimised MPP programming by pressure-volume haemodynamic measurements, and Forleo et al.^8^ by seven different methods. Of the studies evaluating MPP-mixed programming when compared to convention CRT, Niazi et al.^9^ reported no clinical improvement (OR: 0.78, 95% CI: [0.50–1.23], *p* = .291) and Leclercq et al reported no echocardiographic improvement (OR: 0.91, 95% CI: [0.62–1.34], *p* = .647) (Figure 3). A further sub analysis was performed of studies where efficacy of MPP-AS was compared to MPP-Other. Niazi et al provided an improvement in CCS as an endpoint when comparing MPP-AS to MPP-Other, and MPP-AS was more efficacious (OR: 6.79, 95% CI: [2.44–18.90], *p* < .001). Similarly Leclercq et al.^10^ demonstrated greater efficacy with MPP-AS when evaluating a reduction in LVESV >15% (OR: 2.36, 95% CI: [1.31–4.25], *p* = .004). However, only 52 and 68 patients were programmed to MPP-AS in these respective studies, representing a small sample size (Table 3).

### 3.3 Echocardiographic changes

**Figure 4:**
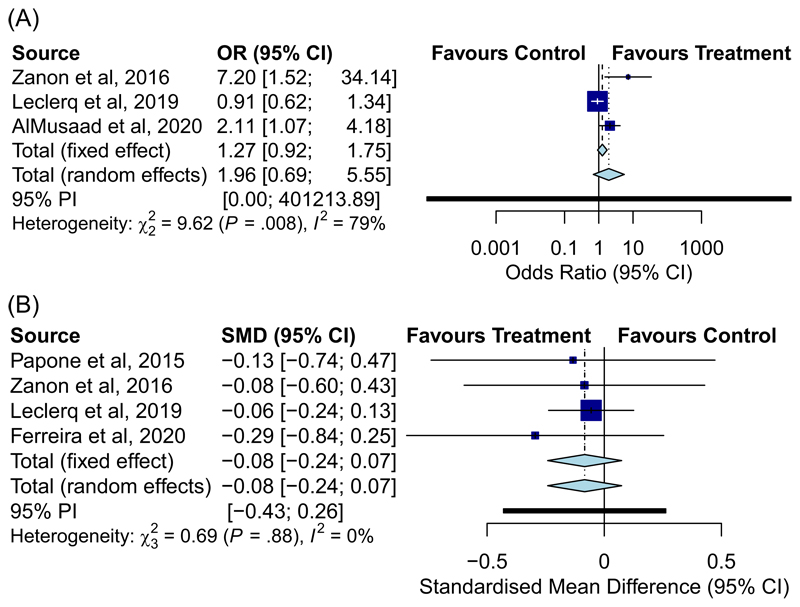
Forest plot displaying odds ratio of reduction in LVESV >15% of reporting studies (A) and reduction in SMD of absolute LVESV value at follow-up (B) for conventional CRT versus MPP. LVESV, left ventricular end systolic volume; SMD, standardised mean distance

Echo response was reported in different ways in the studies. There was no significant difference at follow-up in absolute LVESV value [SMD: −0.084, 95% CI: [−0.24–0.074], *p* = .30; I^2^ = 0%) or absolute EF (SMD: 0.22, 95% CI: [−0.32–0.77], *p* = .42; I^2^ = 75%) when comparing MPP vs conventional CRT in studies that reported these outcomes (Figure 4). Similarly there was no significant difference in LVESV reduction of >15% (OR: 1.96, 95% CI: [0.69–5.55], *p* = .20; I^2^ = 79%). The number of “super-response” cases, as defined as >30% reduction in LVESV following MPP trended towards significance (OR: 2.51, 95% CI: [0.99–6.37], *p* = .054; I^2^ = 48%).

### 3.4 NYHA and CCS change

**Figure 5:**
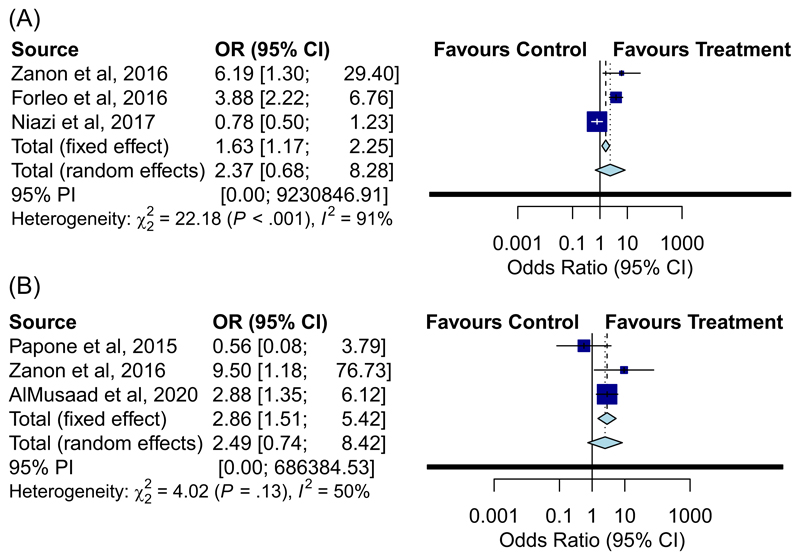
Forest plot displaying odds ratio of improvement in CCS of reporting studies (A) and improvement by at ≥1 NYHA class (B) for conventional CRT versus MPP. CCS, Clinical Composite Score; CRT, cardiac resynchronisation therapy; NYHA, New York Heart Association

CCS was defined as per the descriptions for relevant studies in Table 1. There was no significant difference in improvement in NYHA class of ≥1 (OR: 2.49, 95% CI: [0.74–8.42], *p* = .141; I^2^ = 50%) or improvement in CCS of ≥1 (OR: 2.37, 95% CI: [0.68–8.28], *p* = .178; I^2^ = 91%) (Figure 5).

### 3.5 Sensitivity analysis

In the sensitivity analysis, excluding the abstracts by Al Mussad and Ferreira, resulted in no differences in the odds ratios and significance of results with regards to the prespecified endpoints^20,22^ (Table 4).

## 4 Discussion

The current meta-analysis adds important new data on the role of MPP from a quadripolar lead in the improvement of outcomes of CRT response and represents a critical addition to the literature evaluating the efficacy of MPP. Since the publication of a previous metanalysis by Hu et al there have been two major randomised trials^9,10^ and two further preliminary randomised studies of MPP efficacy published.^20–22^ The prior meta-analysis of Hu et al incorporated smaller, non-randomised studies with a high risk of bias. The current analysis demonstrates superiority of MPP over conventional CRT when evaluating both randomised and nonrandomised studies together. Notably, the benefit of MPP is not significant when including randomised studies only. This is an important finding as MPP activation will adversely affect battery life and therefore, there needs to be a robust clinical reason to consider its activation.^6^ Additionally, not all mechanistic studies have demonstrated benefit with MPP compared to single site optimised LV stimulation.^23,24^

### 4.1 Variability in study design and efficacy outcomes

There was considerable variability and heterogeneity in the studies with differing patient populations and end points that make it difficult to draw firm conclusions as to the therapeutic benefit of MPP. We did not specifically compare the effects of MPP in ischemic and nonischemic patients as there was insufficient data to allow results to be included in the final analysis. Of the studies included, Papone et al identified that ischaemic patients underwent significant improvements in EF, however no improvement at 12 months with respect to LVESV, with MPP compared with conventional CRT, however only 9 patients had an ischaemic aetiology. Leclercq et al.^10^ did not identify any improvement in ischaemic individuals with respect to reduction in LVESV >15% in ischaemic patients (OR: −0.8 [−12.1–10.4]), in a much larger group of 215 patients. Further analysis of this may be important as MPP could be particularly beneficial in this group by avoiding areas of slow conduction or scar.^25,26^ Likewise, we did not compare the effects in patients with LBBB and non-LBBB conduction disorders due to insufficient sub-group data. In addition, final LV lead position was not universally described in the studies included. Of those that did describe this, Leclercq et al did not identify any significant difference in position between groups (*p* = .621); and neither did Zanon et al, who identified similar basal and mid lateral LV lead position proportions between groups. Similarly, Papone et al did not identify a significant difference in final LV lead position, however, there was a tendency to more basal LV position in the conventional CRT group (27% vs. 9%, *p* = .09). MPP was used to treat both CRT naïve patients and those patients that had already been identified as CRT nonresponders and this may have confounded the results. To address this there are ongoing multicentre randomised studies which aim to assess the effect of MPP in previously CRT naïve patients that may address this.^27^

### 4.2 MPP programming

The results of the current analysis suggest a signal towards improved response when MPP is programmed with maximal anatomical separation (MPP-AS). It could be argued that the results of MPP may be dependent on programming and that MPP-AS may capturing a wider area of myocardium, and potentially avoiding scar may result in better electrical synchronization and improved outcomes. In keeping with this hypothesis, the two large published randomised studies of Niazi^9^ and Leclercq^10^ both found a signal for benefit of MPP-AS. It should, however, be noted that in both cases the sub-groups were neither significant in sample size, prespecified, nor randomised, and therefore, we cannot draw firm conclusions from these findings. Based on the initial findings of phase I of the MORE-CRT MPP, where the widest anatomical spacing was not prespecified but programming was left to the discretion of the implanter,^10^ phase II of the MORE-CRT MPP study has been undertaken recruiting over 4000 additional patients in whom MPP-AS is the mandated programming.^28,29^ The results of phase II are not yet available but when published should be able to address the question as to whether MPP-AS results in improved clinical outcomes compared to standard CRT.

### 4.3 Clinical perspective

Since their initial introduction, quadripolar leads have revolutionised CRT with initial studies demonstrating reduced PNS and reintervention.^30^ Subsequent studies have shown cost effectiveness^31^ and mortality benefit,^32^ and as such they have become standard of care. The ability to program vectors from multiple poles of an LV lead has potential advantages in terms of improving CRT response, however initial positive findings in small, single centre acute studies and nonrandomised studies have not, to date, been reflected in larger randomised studies with lower risk of bias. MPP may also have a deleterious effect with a significant reduction in battery longevity. Few studies have specifically assessed the impact of MPP on battery longevity compared to conventional CRT. Of the included studies, Leclercq et al is the only to comment that MPP can reduce battery longevity by approximately 1 year (or approximately 15%), with a separate sub-study of Spanish participants in the MORE-CRT study demonstrated similar reductions in battery capacity of 15 ± 14% in those programmed to MPP-AS.^33^ A sub-analysis of the IRON-MPP study concluded that out of 237 patients followed up, MPP was associated with 0.44 years reduction in projected battery longevity.^34^ In a separate dataset, a small observational study of 46 patients by Akerstrom et al, demonstrated that MPP-AS programming significantly shortened battery longevity compared with conventional CRT at three pacing capture thresholds (≤1.5 V, −5.6%; ≤4.0 V, −16.9%; ≤6.5 V, −21.3%; *p* < <0.001).^35^ This is an important point to consider, due to the increased risk associated with repeated device intervention, as illustrated by the REPLACE registry demonstrating a 4% risk of major complication 6 months following generator change.^36^

It also may not be possible to program MPP in all patients due to high capture thresholds or phrenic nerve stimulation (PNS). Of the included studies that evaluated capture threshold (CT), varying levels of CTs in patients randomised to MPP were identified. Forleo et al reported that whilst 97% of patients were programmable to MPP, this was at a CT of 5 V, however only 87% were programmable at 3 V without PNS. In addition, 4% of patients were not programmable to MPP due to PNS. Niazi et al reported that 3.2% of patients were not programmable to MPP, whilst similar results were noted by Leclercq et al, with only 2.8% not being able to be programmed to MPP due to PNS or high CT.

Given these uncertainties it is difficult to justify the widespread use of MPP programming in all CRT patients and its use may be better restricted to certain groups of patients where there is a good reason to believe that its activation may be beneficial, although based on the current data it is unclear as to which group of patients this may relate to. In addition, alternative technologies including multisite pacing in the form of multilead CRT with an additional right or left ventricular lead may offer an alternative solution to higher rates of nonresponse in certain patient groups. However, published results involve trials with a small number of participants with softer outcomes.^37–39^ It is hoped that further large, randomised studies will help clarify in which patients this treatment may be beneficial and how it should be programmed in those patients.

## 5 Limitations

The current meta-analysis has important limitations. Several the studies were nonrandomised, and observational cohort studies or registries, and such studies have inherent confounders and biases as highlighted in the risk of bias assessment that we performed. In addition, there were two studies which were not fully peer reviewed. As stated in the methodology, the inclusion of so-called “grey literature” is encouraged to avoid selection and publication bias, these should be approached with caution. To mitigate this and justify their inclusion a sensitivity analysis was performed which justified inclusion of grey literature. Notably, the weighting assigned to studies that are fully peer reviewed and are considered high quality were given greater weighting in the final ROB assessment (figure S2). There were multiple different measures of MPP outcome and follow-up duration was not standardised across the studies. This may have affected the outcome, as the marginal benefits of MPP over conventional CRT may require longer therapy duration than the follow-up periods allowed in the current studies. In several studies MPP was assessed only in nonresponders to CRT and in fact, this was the case in the largest major published randomised trial by Leclercq et al.^10^ It could be argued that MPP in this group of patients who have already failed to respond to conventional CRT may not be of benefit, as response is unlikely due to underlying unfavourable substrate in this patient group which is not amenable to CRT.

Furthermore, MPP programming varied across studies, and was often at the discretion of the implanter making it difficult to systematically assess the electrical benefit of MPP and evaluate an individual effect of each programming type. We mitigated this risk by assuming high levels of heterogeneity, and so a random effects meta-analytical approach was applied to all analyses. In the future, as further studies are performed with more detailed information on programming type, a network meta-analysis may be an appropriate way to evaluate types of MPP programming better. Delivery of MPP was via two different models of CRT devices and not consistently personalised to the patient’s cardiac substrate and it is possible that such measures may optimise electrical resynchronisation and improve response. Specifically, ensuring that both pacing sites are outside of scar, may improve response.

## 6 Conclusion

The current meta-analysis represents the largest to date on the use of MPP. There appears to be a signal for benefit for MPP mainly derived from nonrandomised trials, whilst more recent larger randomised studies have failed to show a clear benefit. The results of ongoing large scale randomised studies will be required to better assess the potential benefit of MPP. At present it is difficult to justify wide-spread use of MPP in the CRT population.

## Figures and Tables

**Table 1:** Characteristics of studies included in the final analysis

Author, year	Trial registration number	Study design	Period of active run-in (months)	Total included in analysis (*n*)	Notable inclusion criteria	CRT device and LV lead implanted	Primary efficacy end point	Secondary efficacy end point	Final MPP Programme (MPP-AS/MPP-Other/ MPP-Mixed)
Papone et al. (2015)	NCT01564186	Parallel RCT (with active run-in)	3	42	CRT implant as per ESC/ EHRA guidelines	CRT Device: Unify Quadra MP™ or Quadra Assura MP™, SJM LV Lead: Quartet™ quadripolar lead, SJM	Reduction in LVESV >15%	-LVESV (absolute & relative change) -LVEF (absolute & relative change) -NYHA class -LVEDV (absolute)	MPP-Other
Zanon et al. (2016)	NR	Retrospective observational registry	NA	74	CRT Implant as per ESC/ EHRA guidelines	CRT Device: Quadra Assura MP™, SJM LV Lead: Quartet™ quadripolar lead, SJM	Reduction in LVESV >15%	-ESVi (absolute) -NYHA class -Packer Score	MPP-Other
Forleo et al. (2016)	NCT02606071	Prospective observational registry	NA	232	Any patient following CRT-D implant	CRT Device: Unify Quadra MP™ or Quadra Assura MP™, SJM LV Lead: Quartet™ quadripolar lead, SJM	Clinical Composite Score (Death, hospitalization for HF, increase in NYHA class)	-Increase in LVESV >5%	MPP-Other
Niazi et al. (2017)	NCT01786993	Parallel RCT (with active run-in)	3	381	De novo CRT-D incl. upgrades	CRT Device: Quadra MP™ CRT-D, SJM LV Lead: Quartet™ quadripolar lead, SJM	Clinical Composite Score (All cause mortality, HF-related hospitalization, NYHA class, Patient Global Assessment (PGA) score)	-Nil	MPP-Mixed (MPP-AS & MPP-Other)
Leclercq et al. (2019)	NCT02006069	Parallel RCT (with active run-in)	6	467	CRT implant as per ESC/ EHRA guidelines	CRT Device: Quadra Assura MP™, SJM LV Lead: Quartet™ quadripolar lead, SJM	Reduction in LVESV >15%	-LVESV (absolute & relative) -HF events per 100 patient years	MPP-Mixed (MPP-AS & MPP-Other)
AIMusaad et al. (2020)	NR	Parallel RCT (without active run-in)	NA	142	De Novo CRT-D LBBB Atrial rate >40 bpm Non-AF	CRT Device: Unify Quadra MP™ or Quadra Assura MP™, SJM LV lead: Quartet™ quadripolar lead, SJM	Reduction in LVESV >15%	-Reduction in LVESV >30% -Composite (LVESV >10% and increase in LVEF >5%) -NYHA class -EF (absolute) -QRSd change (%) -LVESV (absolute)	MPP-AS
Ferreira et al. (2020)	NR	Parallel RCT (with active run-in)	6	52	NR	CRT Device: NR LV Lead: Quartet™ quadripolar lead, SJM	Reduction in LVESV >30%	-LVESV (absolute & relative) -LVEF (absolute & relative) -QoL – E5QD, MLWHF, 6MWT -NT-Pro BNP	MPP-AS

Abbreviations: AF, atrial fibrillation; CRT, cardiac resynchronisation therapy; EHRA, European Heart Rhythm Association; E5QD, EuroQol; ESC, European Society of Cardiology; HF, heart failure; LBBB, left bundle branch block; LVEDV, left ventricular end diastolic volume; LVEF, left ventricular ejection fraction; LVESV(i), left ventricular end systolic volume (indexed); MLWHF, Minnesota Living with Heart Failure; MPP, multipoint pacing; 6MWT, six minute walk test; NA, not applicable; NR, not recorded; NT-Pro BNP, N-terminal pro B-type natriuretic peptide; NYHA, New York Heart Failure; QoL, Quality of Life; QRSd, QRS duration; RCT, randomised controlled trial; SJM, St Jude Medical/Abbott.

**Table 2:** Baseline characteristics of participants included by study

Author, year	Age (years)			Ischaemic aetiology (%)			Male (%)			LVEF (%)			LVESV (ml)			LBBB (%)			QRSd (ms)			Follow-up (months)		
	BIP	MPP		BIP	MPP		BIP	MPP		BIP	MPP		BIP	MPP		BIP	MPP		BIP	MPP		NIP	MPP	
Papone et al. (2015)	67 ± 8	66 ± 8		36.0	55.0		73.0	86.0		30 ± 6	28 ± 5		170 ± 104	177 ± 56		59.0	55.0		151 ± 17	153 ± 16		12.0	12.0	
Zanon et al. (2016)	69.7 ± 10.4	67.4 ± 12.5		50.0	55.0		68.5	80.0		30.4 ± 6.3	27.2 ± 4.3		76.6 ± 25.4^a^	72.9 ± 28.2^a^		61.1	65.0		NR	NR		26.5	23.4	
Forleo et al. (2016)	71.0 ± 10.0	69 ± 11.0		47.0	41.0		79.0	81.0		28.1 ± 6.0	28.2 ± 5.9		NR	NR		78.0	71.0		157 ± 23	164 ± 3		6.0	6.0	
Niazi et al. (2017)	68 ± 10	67 ± 10		48.9	47.8		64.8	66.1		NR	NR		NR	NR		77.2	73.4		154 ± 20	158 ± 24		9.0	9.0	
Leclercq et al. (2019)	68 ± 10	68 ± 11		51.5	56.4		79.7	80.9		26 ± 8	26 ± 8		NR	NR		65.1	68.4		156 ± 25	157 ± 25		12.0	12.0	
AIMusaad et al. (2020)	59 (IQR: 52.1–67.0]	62.1 (IQR: 52.3–69.4)		30.4	35.6		69.6	65.8		25.7 (IQR: 21.7–32.5)	26.0 (IQR: 21.1–33.6)		140 (IQR: 102–191)	130 (IQR: 103–206)		100.0	0.0		158 (IQR: 150.0–170.0)	160 (IQR: 150–172.3)		6.0	6.0	
Ferreira et al. (2020)	NR	NR		NR	NR		NR	NR		37.1 ± 12.0	38.3 ± 9.8		92.2 ± 47.3	93.4 ± 52.3		NR	NR		NR	NR		12.0	12.0	

Abbreviations: IQR, interquartile range; LBBB, left bundle branch block; LVEF, left ventricular ejection fraction; LVESV, left ventricular end systolic volume; NR, not recorded; QRSd, QRS duration.

a LVESV is recorded as ml/m^2^.

**Table 3:** Table demonstrating odds ratio of achieving reported efficacy endpoint in studies which evaluated MPP-AS versus MPP-other programming

Author, year	Efficacy endpoint evaluated	No. of MPP-AS patients	No. of MPP-other patients	OR (95% CI)	*p* value
Niazi et al. (2017)	CCS improvement by >1	52	37	6.79 (2.44–18.90)	<.001
Leclerq et al. (2019)	Reduction in LVESV >15%	68	168	2.36 (1.31–4.25)	.004

Abbreviations: CCS, clinical composite score; CI, confidence interval; LVESV, left ventricular end systolic volume; OR, odds radio.

**Table 4:** Sensitivity analysis based on publication status

Endpoint	Calculation method	Including abstract and preprint (7 studies)	Excluding abstract and preprint (5 studies)
		OR (95% CI)	OR (95% CI)
Pooled echocardiographic response	Random effects	2.70 (1.38–5.30)	2.70 (1.01–7.20)
Pooled clinical response	Random effects	2.40 (0.89–4.71)	1.85 (0.62–5.51)
Reduction in LVESV >15%	Random effects	1.96 (0.69–5.55)	2.22 (0.30–16.48)
Clinical Composite Score	Random effects	2.42 (0.99–5.90)	2.37 (0.68–8.28)
Improvement by at ≥1 NYHA class	Random effects	2.49 (0.74–8.42)	2.23 (0.14–35.70)
		SMD (95% CI)	SMD (95% CI)
LVESV change (absolute)	Random effects	− 0.08 (−0.24–0.07)	− 0.06 (−0.023–0.10)

Abbreviations: CI, confidence interval; OR, odds ratio; SMD, standardised mean difference.
